# Stathmin and phospho-stathmin protein signature is associated with survival outcomes of breast cancer patients

**DOI:** 10.18632/oncotarget.4276

**Published:** 2015-06-13

**Authors:** Xia-Ying Kuang, Li Chen, Zhi-Jie Zhang, Yi-Rong Liu, Yi-Zi Zheng, Hong Ling, Feng Qiao, Shan Li, Xin Hu, Zhi-Ming Shao

**Affiliations:** ^1^ Key Laboratory of Breast Cancer in Shanghai, Department of Breast Surgery, Fudan University Shanghai Cancer Center, Shanghai, China; ^2^ Department of Oncology, Shanghai Medical College, Fudan University, Shanghai, China; ^3^ Institutes of Biomedical Science, Fudan University, Shanghai, China; ^4^ Department of Epidemiology and Biostatistics, School of Public Health, Fudan University, Shanghai, China

**Keywords:** stathmin, phosphorylation, breast cancer, prognostic model, paclitaxel

## Abstract

Currently, Stathmin1 (STMN1) and phospho-STMN1 levels in breast cancers and their clinical implications are unknown. We examined the expression of STMN1 and its serine phospho-site (Ser16, Ser25, Ser38, and Ser63) status by immunohistochemistry. Using Cox regression analysis, a STMN1 expression signature and phosphorylation profile plus clinicopathological characteristics (STMN1-E/P/C) was developed in the training set (*n* = 204) and applied to the validation set (*n* = 106). This tool enabled us to separate breast cancer patients into high- and low-risk groups with significantly different disease-free survival (DFS) rates (*P* < 0.001). Importantly, this STMN1-E/P/C model had a greater prognostic value than the traditional TNM classifier, especially in luminal subtype breast cancer (*P* = 0.002). Further analysis showed that patients in the low-risk group would benefit more from adjuvant paclitaxel-based chemotherapy (*P* = 0.002). In conclusion, the STMN1-E/P/C signature is a reliable prognostic indicator for luminal subtype breast cancer and may predict the therapeutic response to paclitaxel-based treatments, potentially facilitating individualized management.

## INTRODUCTION

Breast cancer is the most frequently diagnosed cancer and the leading cause of cancer-related morbidity and mortality among women worldwide [1]. As a heterogeneous disease, comprehensive gene expression profiling has distinguished four major molecular subtypes of breast cancer with different clinical outcomes: luminal A, luminal B, HER2/neu and triple-negative [2–4]. The luminal A and B subtypes are collectively referred to as the luminal type, which accounts for 65–70% of breast cancers. Compared with other breast cancers, patients with luminal subtypes benefit from endocrine therapies and have a better prognosis. However, long-term recurrence remains a major clinical problem. Additional markers to individualize treatment and prognosis are urgently needed.

Stathmin (STMN1), also known as Oncoprotein 18 (Op 18), is a ubiquitous, highly conserved 18-kDa cytosolic phosphoprotein which increases the rate of mitosis through upregulation of microtubule dynamics [5]. STMN1 has 4 serine phosphorylation sites (Ser16, Ser25, Ser38, and Ser63). Phosphorylation at either Ser16 or Ser63 strongly reduces or abolishes the ability of STMN1 to bind to and sequester soluble tubulin [6], while phosphorylation at Ser38 may be a novel biomarker of increased tumor cell proliferation and impaired prognosis [7]. STMN1 Ser16 can be phosphorylated by protein kinase C (PKC), PAK1, or Ca^2+^/calmodulin-dependent kinase II/IV [8–11], whereas Ser25 and Ser38 are targeted by mitogen-activated protein kinases (MAPKs) and cyclin-dependent kinases (CDKs) [12, 13]. Multisite phosphorylation of STMN1 generates different combinations of STMN1 phosphoisomers that contribute to the overall regulation of cell invasion and cancer metastasis. However, the prognostic impact and possible clinical value of STMN1 phosphorylation has not been extensively studied in human cancers, though the impact of phosphorylation at different STMN1 phospho-sites has been explored in some experimental models, primarily in relation to the effects on microtubule formation, proliferation, cell migration, and cancer invasion [6, 14, 15].

To our knowledge, although adjuvant chemotherapy could effectively reduce the risk of metastasis and mortality for women with operable disease, only a fraction of patients benefit from this treatment. Paclitaxel, a microtubule-stabilizing drug, is a common cytotoxic agent that has been used extensively in recent years. However, resistance to paclitaxel is a major obstacle in anticancer treatment. Therefore, we explored the possible interaction between STNM1 phosphorylation status and breast cancer response towards paclitaxel-based adjuvant chemotherapy.

In this study, we evaluated the protein levels of STMN1 and its phosphorylated forms in primary breast cancer specimens to develop a STMN1-based classifier to predict disease-free survival (DFS) for breast cancer patients using a multivariate Cox proportional hazards model with two independent cohorts. We also assessed the prognostic accuracy of this classifier in various breast cancer molecular subtypes. Additionally, we compared its prognostic and predictive accuracy against traditional clinicopathological risk factors, and investigated the classifier's predictive value for patient benefit from adjuvant chemotherapy in breast cancer.

## RESULTS

### STMN1 expression and serine phosphorylation in breast cancer patients

**Figure 1:**
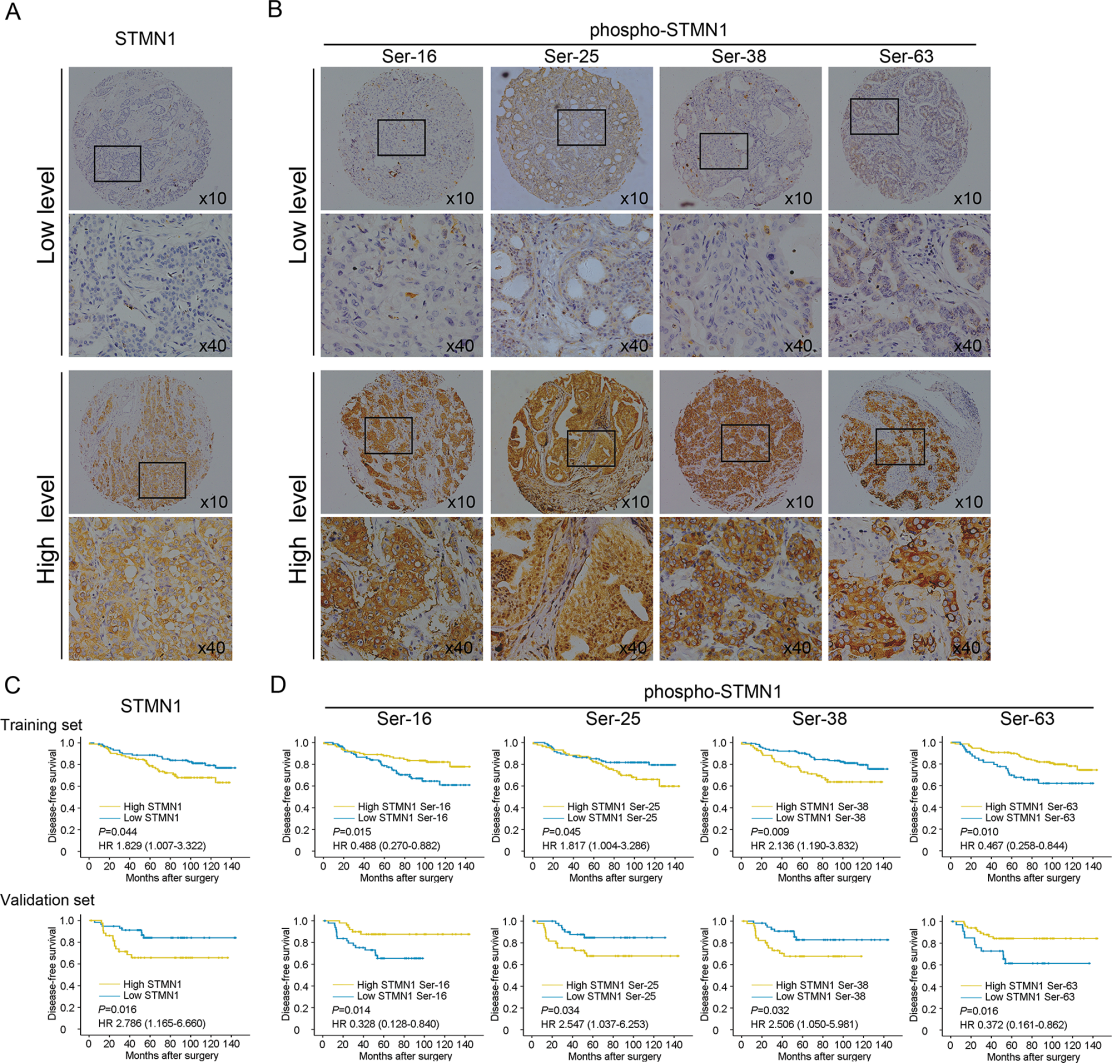
Identification by immunohistochemistry (IHC) of STMN1 and its multiple phosphor-sites in the primary tumor and Kaplan-Meier analysis of DFS in breast cancer patients with high or low STMN1 expression and the expression of its multiple serine phospho-sites **A.** Representative IHC staining of high and low expression of STMN1 in the large (400×) and small images (100×). **B.** Representative IHC staining of high and low expression of multiple phosphor-sites (Ser-16, Ser-25, Ser38, Ser63) in the large (400×) and small images (100×). **C.** Kaplan-Meier analysis of DFS in the training set. **D.** Kaplan-Meier analysis of DFS in the validation set.

In the training set, the median age of the 204 patients was 51 years (range 29–84 years), and the median follow-up time was 102 months (range 0.5–144 months). In the validation set, the median age of the 106 patients was 55 years (range 34–85 years), and the median follow-up time was 79 months (range 2–81 months). 25 Cases which either lacked follow-up data or experienced tissue loss after IHC staining were excluded. 68 patients experienced distant metastasis in the two sets (Table 1). Immunohistochemical staining for STMN1 (145/310, 46.8% positive), Ser16 (156/310, 50.3% positive), Ser25 (146/310, 47.1% positive), Ser38 (122/310, 39.4% positive), Ser63 (207/310, 66.8% positive) was reviewed and analyzed by two individual pathologists for both sets (Figure 1).

**Table 1:** Characteristics of breast cancer patients in the two sets

Characteristics	Training set (*n* = 204)	Validation set (*n* = 106)	*P*^a^
	No.	No.	
**Median age (range)**	51 y (29–84)	55 y (34–85)	0.413
**Median follow-up time (range)**	102 mo (0.5–144)	79 mo (2–81)	**0.041**
**Age**			
<50 y	91 (44.6%)	52 (49.1%)	0.273
≥50 y	113 (55.4%)	53 (50%)	
NA	0 (0.0%)	1 (0.9%)	
**Menopausal status**			0.478
Premenopausal	84 (41.2%)	46 (43.4%)	
Postmenopausal	120 (58.8%)	60 (56.6%)	
**Tumor stage**^b^			0.298
I	59 (28.9%)	32 (30.2%)	
II	107 (52.5%)	47 (44.3%)	
III	37 (18.1%)	26 (24.5%)	
NA	1 (0.5%)	1 (0.9%)	
**Histological grade**^b^			0.994
I & II	125 (61.3%)	26 (24.5%)	
III	85 (41.7%)	80 (75.5%)	
**Tumor size**			0.448
<2 cm	93 (45.6%)	49 (46.2%)	
≥ 2 cm	111 (54.4%)	57 (53.8%)	
**Node status**			0.901
Negative	117 (57.4%)	61 (57.5%)	
Positive	92 (45.1%)	45 (42.5%)	
NA	1 (0.5%)	0 (0.0%)	
**ER status**			0.301
Negative	94 (46.1%)	59 (55.7%)	
Positive	113 (55.4%)	46 (43.4%)	
NA	3 (1.5%)	1 (0.9%)	
**PR status**			0.294
Negative	91 (44.6%)	66 (62.3%)	
Positive	115 (56.4%)	39 (36.8%)	
NA	4 (2.0%)	1 (0.9%)	
**HER2 status**			**<0.001**
Negative	104 (51.0%)	83 (78.3%)	
Positive	98 (48.0%)	22 (20.8%)	
NA	2 (1.0%)	1 (0.9%)	
**Chemotherapy**			**<0.001**
Paclitaxol-based	14 (6.9%)	29 (27.4%)	
Non-paclitaxol-based	176 (86.3%)	61 (57.5%)	
No chemotherapy	14 (6.9%)	16 (15.1%)	

Abbreviations: ER, estrogen receptor; PR, progesterone receptor; HER2, human epidermal growth factor receptor 2; NA, not available

Bold values are significant (*P* < 0.05).

a Compared using Student's *t* test or Pearson's χ^2^ test.

b Classified according to the National Comprehensive Cancer Network guidelines.

The correlations between patients' clinical characteristics and levels of total STMN1 and phospho-STMN1 forms are summarized in Supplementary Table S1. No significant differences were observed between the markers and clinicopathological characteristics.

### STMN1 expression and its serine phosphorylation status is associated with DFS in breast cancer

Kaplan-Meier analysis showed that STMN1, Ser25 and Ser38 were strongly associated with poorer DFS (*P* = 0.044 for STMN1, *P* = 0.045 for Ser25, *P* = 0.009 for Ser38), whereas Ser16 and Ser63 were associated with better DFS (*P* = 0.015 for Ser16, *P* = 0.010 for Ser63). Our analysis in the validation cohort displayed a similar trend for these associations (*P* = 0.016 for STMN1, *P* = 0.014 for Ser16, *P* = 0.034 for Ser25, *P* = 0.032 for Ser38, and *P* = 0.016 for Ser63, Figure 1). As shown in Table 2, both univariate and adjusted multivariate survival analyses revealed a significant difference between the positive- and negative-staining groups for each marker. In the training cohort, cases with high STMN1 expression had a higher likelihood of disease events (HR = 1.829, 95% CI: 1.007–3.322, *P* = 0.047). Phosphorylation at Ser25 (HR = 1.817, 95% CI: 1.004–3.286, *P* = 0.048) and Ser38 (HR = 2.136, 95% CI: 1.190–3.832, *P* = 0.011) were also prognostic factors for poor DFS. In contrast, phosphorylation at Ser16 (HR = 0.488, 95% CI: 0.270–0.882, *P* = 0.018) and Ser63 (HR = 0.467, 95% CI: 0.258–0.844, *P* = 0.012) were tightly associated with improved DFS. In the validation set, we found similar trends with poor DFS for STMN1 (HR = 2.786, 95% CI: 1.165–6.660, *P* = 0.021), phosphorylation at Ser25 (HR = 2.547, 95% CI: 1.037–6.253, *P* = 0.041) and phosphorylation at Ser38 (HR = 2.506, 95% CI: 1.050–5.981, *P* = 0.038), whereas phosphorylation at Ser16 (HR = 0.328, 95% CI: 0.128–0.840, *P* = 0.020) and Ser63 (HR = 0.372, 95% CI: 0.161–0.862, *P* = 0.021) were correlated with prolonged DFS in breast cancer patients.

**Table 2:** Univariate association of the STMN1-E/P model, clinicopathological characteristics, and single phospho-sites status with disease-free survival

	Training set (*n* = 204)		Validation set (*n* = 106)	
	HR (95% CI)	*P* value	HR (95% CI)	*P* value
Age	1.322 (0.731–2.391)	0.356	0.470 (0.197–1.122)	0.089
Menopausal status	1.495 (0.862–2.592)	0.152	0.742 (0.322–1.713)	0.485
Histological grade	2.038 (1.119–3.710)	**0.020**	2.058 (1.076–4.817)	**0.046**
Tumor size (<2 cm vs. ≥2 cm)	1.743 (1.091–2.784)	**0.020**	3.388 (1.450–7.919)	**0.005**
Node status	2.410 (1.326–4.379)	**0.004**	3.516 (1.517–8.154)	**0.003**
ER status	0.916 (0.513–1.635)	0.767	0.544(0.221–1.337)	0.184
PR status	0.733 (0.379–1.417)	0.356	0.455 (0.167–1.237)	0.123
HER2 status	0.893 (0.499–1.595)	0.701	1.261 (0.493–3.223)	0.628
STMN1	1.829 (1.007–3.322)	**0.047**	2.786 (1.165–6.660)	**0.021**
Ser16	0.488 (0.270–0.882)	**0.018**	0.328 (0.128–0.840)	**0.020**
Ser25	1.817 (1.004–3.286)	**0.048**	2.547 (1.037–6.253)	**0.041**
Ser38	2.136 (1.190–3.832)	**0.011**	2.506 (1.050–5.981)	**0.038**
Ser63	0.467 (0.258–0.844)	**0.012**	0.372 (0.161–0.862)	**0.021**
STMN1-E/P model risk score^a^	3.029 (1.599–5.737)	**0.001**	3.736 (1.378–10.129)	**0.010**
STMN1-E/P/C model risk score^b^	6.792 (3.159–14.604)	**<0.001**	4.371 (1.478–12.930)	**0.008**

a A risk model based on STMN1 expression and its multiple phospho-sites status

b STMN1-E/P/C model plus clinicopathological features

### Development of a prognostic signature using combined STMN1 expression and serine phosphorylation status for breast cancer patients

A Cox proportional hazards model was used to build a prognostic classifier [16], which included STMN1 expression and the phosphorylation status of the four phospho-serine sites identified in the training cohort. Here, we derived a formula to calculate a score for metastatic risk in terms of DFS for each patient based on the individual status of those five markers, where risk score = 0.251*STMN1–0.497*Ser16+0.701*Ser25+0.594*Ser38–0.534*Ser63. In this formula, low expression levels of STMN1 and low phosphorylation levels of phosphorylation at the serine sites are equal to 0, and high levels are equal to 1.

**Figure 2:**
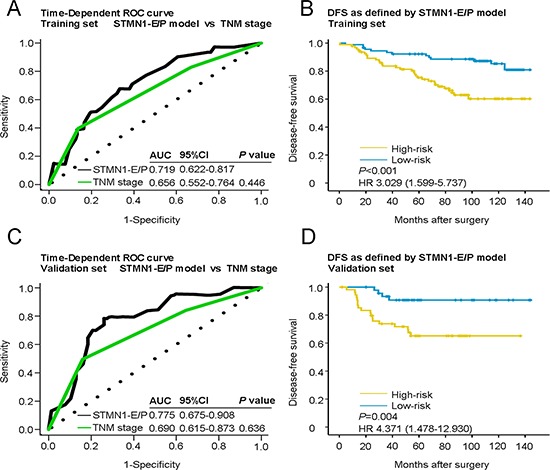
Time-dependent ROC curves for the prognosis of breast cancer by the STMN1-E/P model and Kaplan-Meier survivals in the training and validation sets Data are shown as AUC (95% CI) or hazard ratios (95% CI). ROC = receiver operator characteristic. AUC = area under the curve. **A.** Comparisons of the prognostic accuracy by the STMN1-E/P model and TNM stage in the training set. **B.** DFS of patients with high- or low-risk scores in the training set. **C.** Comparisons of the prognostic accuracy by the STMN1-E/P model and TNM stage in the validation set. **D.** DFS of patients with high- or low-risk scores in the validation set. *P* values were calculated using the log-rank test.

Based on this STMN1 expression and phosphorylation (STMN1-E/P) model, we assessed the prognostic accuracy of the risk score with a time-dependent ROC analysis, it trended towards a higher prognostic accuracy than TNM staging, a traditional prognostic classifier for cancer patients (AUC for STMN1-E/P model: 0.719; AUC for TNM staging: 0.658; Figure 2A). To generate the optimum cutoff score, we used Youden index based on the ROC curve, and chose 0 as the best cutoff risk score [17]. Thus, we classifieded the patients with a risk score of 0 or higher into the high-risk group, and those with a risk score lower than 0 were classified into the low-risk group. By assessing the risk score distribution and DFS status, we found that patients in the low-risk group generally had better survival than the high-risk group (HR = 3.029, 95% CI: 1.599–5.737, *P* < 0.001; Figure 2B). By using Pearson χ^2^ test, several clinicopathological factors, including histological grade, tumor size and lymphatic metastasis, were tightly associated with the STMN1-E/P model driven risk score in the training cohort (Supplementary Table S2).

To confirm the prognostic value of the STMN1-E/P model, the model was applied to the validation set of 106 patients to predict the risk of metastasis for each patient (Table 1). It also had a better prognostic value than TNM staging (AUC for STMN1-E/P model: 0.775; AUC for TNM stage: 0.698; Figure 2C). In addition, the Kaplan-Meier analysis showed a significant difference between the high- and low-risk groups in the validation cohort (HR = 4.371, 95% CI: 1.478–12.930, *P* = 0.004; Figure 2D).

**Figure 3:**
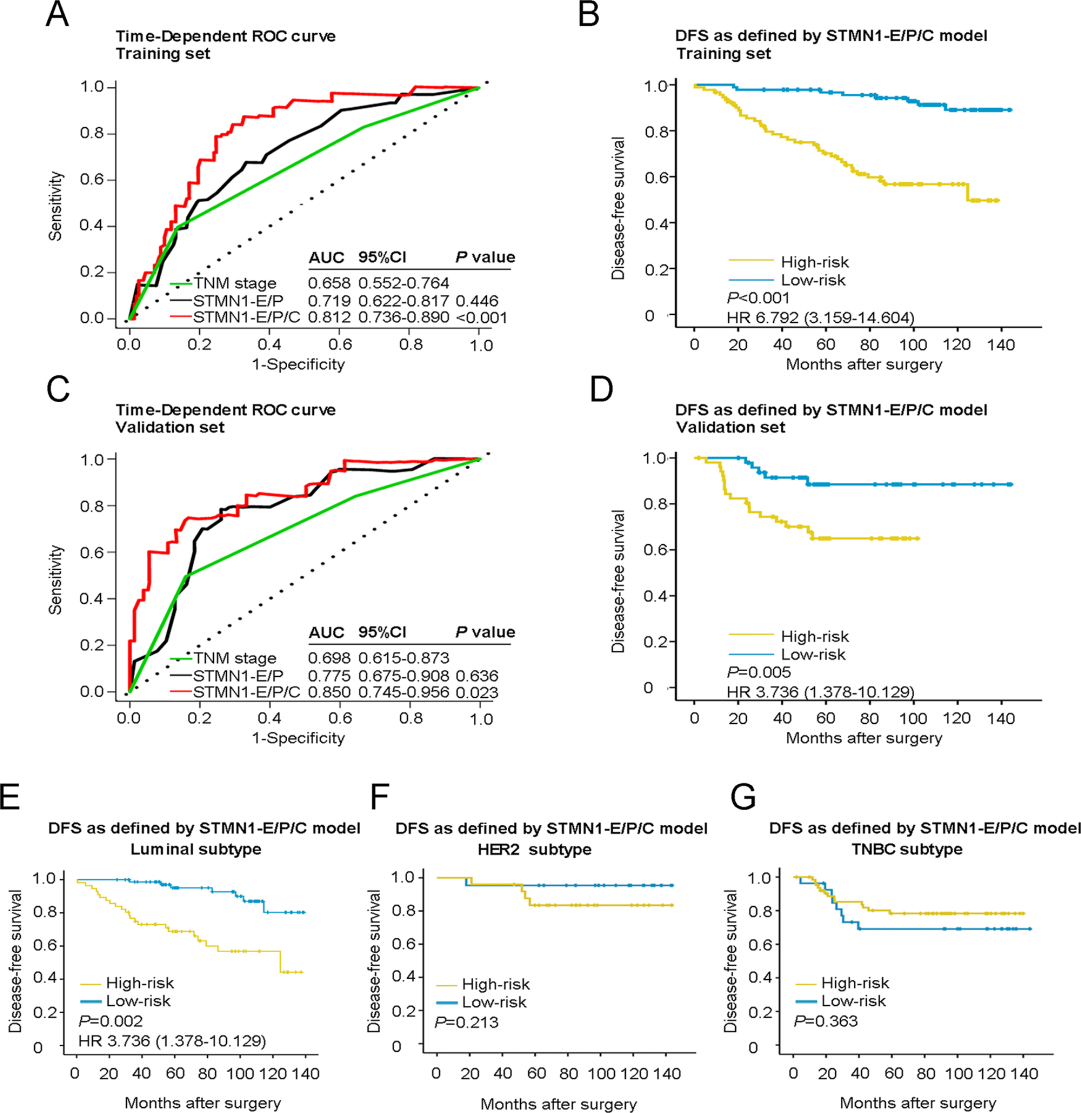
Time-dependent ROC curves for prognosis of breast cancer by the STMN1-E/P/C model and Kaplan-Meier survivals in patients of two sets and different subtypes of breast cancer with high- or low-risk according to the STMN1-E/P/C model **A.** Comparisons of the prognostic accuracy by the STMN1-E/P/C model, STMN1-E/P model and TNM stage in the training set. **B.** DFS of patients with high- or low-risk scores according to the STMN1-E/P/C model in the training set. **C.** Comparisons of the prognostic accuracy by the STMN1-E/P/C model, STMN1-E/P model and TNM stage in the validation set. **D.** DFS of patients with high- or low-risk scores according to the STMN1-E/P/C model in the validation set. *P* values were calculated using the log-rank test. **E.** DFS of patients with luminal breast cancer. **F.** DFS of patients with HER2/neu subtype breast cancer. **G.** DFS of patients with TNBC subtype breast cancer.

To further optimize this classifier, all pathological variables and biological markers were subjected to a univariate analysis. Eight separate prognostic factors emerged: STMN1, Ser16, Ser25, Ser38, Ser63, histological grade, tumor size and lymphatic metastasis. Multivariate Cox model analysis indicated that these factors were also independent predictors of breast cancer metastasis. Using the multivariate Cox proportional hazards model, we calculated a new risk score for individuals to include these factors, where risk score = 0.173*STMN1–0.608*Ser16+0.743*Ser25+0.685* Ser38–0.342*Ser63+0.459*histological grade+0.448*tumor size+0.946*lymphatic metastasis. This new risk score shows an even better prognostic value than the TNM stage both in the training set and the validation set (AUC for STMN1-E/P/C model: 0.812; AUC for TNM stage: 0.658, *P* < 0.001 for the training set; AUC for STMN1-E/P/C model: 0.850; AUC for TNM stage: 0.698, *P* = 0.023 for the validation set) (Figure 3A and 3C). We defined a cutoff score of 2.2 by ROC analysis for this STMN1-E/P/C model. The risk score was tightly associated with a group of well-known clinical features (Supplementary Table S3). Further Kaplan-Meier analysis showed that this novel STMN1-E/P/C model was able to identify a significant difference in clinical outcome between the high- and low-risk groups in both the training set (HR = 6.792, 95% CI: 3.159–14.604, *P* < 0.001; Figure 3B) and the validation set (HR = 3.736, 95% CI: 1.378–10.129, *P* = 0.005; Figure 3D).

### STMN1-E/P/C model powerfully predicts DFS for patients with Luminal subtype breast cancer

We analyzed the association between DFS and the STMN1-E/P/C model-driven risk score in patient groups stratified by breast cancer molecular subtypes. Interestingly, the risk score only had a perfect prognostic value in the luminal subtype, where patients in the high-risk group had a poorer DFS than the low-risk group (HR = 3.736, 95% CI: 1.387–10.129, *P* = 0.002). No significant differences in DFS were observed between the high- and low-risk groups in the HER2 enrichment and triple-negative subtypes (*P* = 0.213 for Her2-positive and *P* = 0.363 for TNBC; Figure 3E–3G). These findings suggested that the risk score of the STMN1-E/P/C model had the greatest prognostic value specifically for the luminal subtype of breast cancer.

### The STMN1-E/P/C model powerfully predicts response to adjuvant chemotherapy

Adjuvant chemotherapy is crucial for most breast cancer patients. To investigate the association between patient classification by the STMN1-E/P/C model and response to adjuvant chemotherapy, we analyzed the interaction between the model and resistance to chemotherapy in a two-step multivariate analysis. In the first step, the Cox regression model included established prognostic factors but not the risk score (see details in note of Table 3). We found that large tumor size (HR = 1.795, 95% CI: 1.206–2.672, *P* = 0.004), positive lymph node status (HR = 1.485, 95% CI: 1.155–1.909, *P* = 0.002), positive PR (HR = 0.456, 95% CI: 0.220–0.947, *P* = 0.035), and grouping of the risk score (HR = 5.332, 95% CI: 2.682–10.599, *P* < 0.001) were significant independent factors for DFS after multivariate adjustment. In the second step, the interaction between risk score groups and chemotherapy was investigated, along with adjustment for the factors identified in the first step (factors with *P* < 0.10). The risk score from the STMN1-E/P/C model was not associated with response to general adjuvant chemotherapy (*P* = 0.078). Paclitaxel, an anti-microtubule drug, has been widely used as an adjuvant chemotherapy after surgery, and recent studies have suggested that STMN1 overexpression is associated with paclitaxel resistance [18]. We therefore selected patients treated with paclitaxel to determine whether the STMN1-E/P/C model could predict response to the drug. The interaction between the risk score and paclitaxel-based chemotherapy strongly impacted DFS (*P* = 0.002; Table 3). The interaction implies that high-risk patients received only 28% (1/3.532, where 3.532 is the HR of interaction between the risk score and paclitaxel-based chemotherapy) of the benefit from adjuvant paclitaxel-based chemotherapy compared with low-risk patients.

**Table 3:** Multivariate Cox model (DFS) including interaction of adjuvant chemotherapy and grouping of the risk score

Usage of chemotherapy	Factors^a^	*P*	HR (95% CI)
General	Chemo. (no vs. yes)	0.075	0.932 (0.119–3.325)
	Grouping (low-risk vs. high-risk)	<0.001	5.338 (2.699–10.558)
	Interaction, Chemo*Grouping	0.078	1.843 (0.933–3.642)
Paclitaxel-based	Chemo. (no vs. yes)	0.011	3.124 (1.536–6.354)
	Grouping (low-risk vs. high-risk)	<0.001	2.410 (1.326–4.379)
	Interaction, Chemo*Grouping	0.002	3.532 (1.577–7.913)

Abbreviations: Chemo, chemotherapy

NOTE: Multivariate analysis of interaction was conducted in two steps. In the first step, the Cox regression model included established prognostic factors (age, menopausal status, lymph node status, tumor size, histological grade, ER, PR, HER2, grouping of the risk score) but not chemotherapy. The first step demonstrated that tumor size (*P* = 0.004), lymph node status (*P* = 0.002), PR (*P* = 0.035), and grouping of the risk score (*P* < 0.001) were significant independent factors for DFS. Menopausal status (*P* = 0.070) and histological grade trended (*P* = 0.078) towards significance. In the second step, interactions between usage of chemotherapy (general or paclitaxel-based) and grouping of the risk score were investigated along with adjustments for those factors (with *P* < 0.10) identified in the first step. This table shows the results of the second step.

a Here, we present only three items: usage of chemotherapy, grouping of the risk score and the interaction between them. Other parameters (tumor size, lymph node status, PR status, menopausal status and histological grade) are not shown.

## DISCUSSION

STMN1 is a prognostic marker for various types of cancer, such as breast and endometrial cancer [19, 20], and has also been shown to be independent of other factors, such as age, menopausal status, nodal status, nuclear grade, tumor size, and ER, PR, and HER2 expression [21]. We hypothesized that the cellular phosphorylation levels of STMN1 at four serine residues, as determined by immunohistochemistry, are related to DFS in breast cancer. We also sought to explore the possibility that differences in phosphorylation status could prognose recurrence risk in breast cancer patients. By analyzing two independent patient cohorts, we showed and validated for the first time that integrated STMN1 expression, phosphorylation status and known clinicopathological characteristics of cancer patients (shown here for breast cancer) can predict clinical outcomes successfully, and exhibit a remarkable predictive value to guide appropriate treatment.

In this study of breast cancer, STMN1 expression and the phosphorylation status of its multiple serine residues were better correlated with DFS than standard clinicopathological features. Because the DFS differed depending on the site of phosphorylation detected, we expected that the combined data from the four sites would have a higher prognostic value. We developed a novel prognostic model based on STMN1 and its multiple phospho-sites, in addition to several clinicopathological factors, to improve the prognosis of recurrence after surgery for breast cancer patients. We demonstrated that this model can successfully categorize breast cancer patients into high- and low-risk groups with significant differences in DFS, especially for the luminal subtype. Furthermore, this classifier can be utilized to predict the therapeutic response to adjuvant paclitaxel-based chemotherapy. Our findings are based on two independent patient cohorts and indicate that this novel classifier may be useful for managing patients with breast cancer and identifying patients with higher risk for recurrence.

The TNM staging system has been used for many years as a predictor for breast cancer recurrence. However, TNM staging has been gradually shown to exhibit less sensitivity in predicting patient outcome [22]. The recent discovery of aberrant expression levels of various biomarkers, including microRNAs, long-noncoding RNAs, and proteins, in breast cancer tissue or patients' plasma has motivated the analysis of clinical characteristics and biomarkers for breast cancer diagnosis. In this study, we suggest that the risk score based on STMN1 and its multiple phospho-sites could be of significantly better prognostic value than TNM staging.

The addition of some significant clinicopathological factors, including histological grade, tumor size and lymphatic metastasis, to the STMN1-E/P model enhanced the prediction of DFS. Our report is the first in our knowledge to identify the importance of the combination of STMN1 and its multiple phosphorylations in breast cancer. Furthermore, the IHC readouts in the lab plus the clinicopathological factors could accurately predict the probability for breast cancer recurrence, especially for luminal subtype.

Most breast cancer patients are of the luminal subtype [23], with a long-term clinical course and sensitivity to endocrine therapy [24]. However, high probabilities of recurrence long after surgery (more than 5 years) for the luminal subtypes remain a large clinical problem for physicians. Our research establishes the STMN1-E/P/C model as a better predictor of DFS for patients with breast cancer, specifically the luminal subtype. Although our results predicted no significant differences, the time-dependent ROC curves still display a trend towards a better prognostic value for the classifier for longer DFS (Supplementary Figure S1). Further research with longer follow-up times could strengthen the prognostic model for the luminal subtype.

Paclitaxel is one of the most commonly used chemotherapy agents for breast cancer [25] and is also considered one of the most efficacious [26]. Paclitaxel stabilizes microtubules, arresting mitosis [26]. STMN1 has been reported to increase sensitivity to anti-microtubule drugs. Overexpression of stathmin has been shown to decrease polymerization of microtubules and markedly decrease the binding of and sensitivity to paclitaxel but not affect sensitivity to chemotherapeutic drugs that do not target microtubules [27]. Its overexpression has also been associated with paclitaxel resistance in other malignant tumors, including cholangiocarcinoma, lung cancer and ovarian cancer [18, 28, 29]. In the present study, we showed that patients classified as high risk by the STMN1-E/P/C model derive less benefit from paclitaxel; further use of this classifier may better identify patients most likely to benefit from adjuvant paclitaxel-based therapy. The STMN1-E/P/C model is both prognostic and predictive for patients with breast cancer, in that low risk patients have less likelihood of metastasis and a clear benefit from paclitaxel.

In conclusion, we showed that STMN1 expression and phosphorylation at multiple serine residues plus clinicopathological characteristics have significant predictive value for breast cancer-associated disease events including recurrence. This model could improve the identification of patients with luminal subtypes of breast cancer at the time of primary diagnosis and predict their sensitivity to paclitaxel, thereby enabling oncologists to target those likely to relapse or metastasize for appropriate treatment.

## MATERIALS AND METHODS

### Patients and clinical database

We studied 310 formalin-fixed and paraffin-embedded (FFPE) tissue samples from 310 patients with histologically confirmed stage I to III primary breast cancer who underwent mastectomy in the Department of Breast Surgery in Fudan University Shanghai Cancer Center. All specimens were routinely fixed in 10% formalin and embedded in paraffin, and the specimens were selected to represent all of the histologic types of breast cancer. For the training set, data were obtained from 204 patients between August 2001 and March 2006 for whom clinicopathological characteristics and follow-up information were available. The patients of this training set were regularly followed up through September 2013, and the clinical outcomes of 183 cases were obtained with a median follow-up of 102 months (0.5–144 months). We added another 106 patients with the same entry criteria between June 2007 and November 2011 as an independent validation set. The clinical outcomes of 102 cases through July 2014 were obtained with a median follow-up time of 79 months (2–81 months). Patient information and the clinicopathological characteristics of both cohorts are presented in Table 1. This study was approved by the independent ethical committee/institutional review board of Fudan University Shanghai Cancer Center (Shanghai Cancer Center Ethical Committee). All patients provided written informed consent before their inclusion in this study.

### Tissue microarray

To construct the tissue microarray (TMA), hematoxylin and eosin (HE)-stained slides from tumors were evaluated to identify representative tumor regions from which 2 1.0-mm tissue cores were retrieved and transferred into recipient array blocks using a tissue micro arrayer (UNITMA Instruments, Seoul, Korea) as previously described [30, 31]. The TMA was composed of duplicate cores from different areas of the same tumor to compare staining patterns. TMA sections of 3 mm were subsequently dewaxed in xylene and rehydrated in ethanol for immunohistochemical staining.

### Immunohistochemistry

Immunohistochemistry for STMN1, Ser16, Ser25, Ser38 and Ser63 were conducted using a two-step protocol (GTVision™III). Briefly, TMA sections were washed with phosphate-buffered saline (PBS) after rehydration and then treated with 3% hydrogen peroxide for 10 min to block endogenous peroxidase activity. The antigens were retrieved by boiling the five groups of TMAs in citrate buffer (pH 6.0) at 100°C for 5 min for STMN1 and Ser38, 121°C for 10 min for Ser16 and Ser63, and 140°C for 25 min for Ser25. For STMN1 and Ser38, TMAs were blocked with 10% normal goat serum for 1 h at room temperature (RT) and incubated in a humid chamber at 4°C overnight with polyclonal rabbit anti-human STMN1 antibody (Proteintech) or polyclonal rabbit anti-human STMN1 Ser38 (Cell Signaling Technologies) antibody diluted to 1:400 or 1:100, respectively. For the other three markers, TMAs were incubated with polyclonal rabbit anti-human STMN1 Ser16 antibody (Abcam), polyclonal rabbit anti-human STMN1 Ser25 antibody (Novus), and polyclonal rabbit anti-human STMN1 Ser63 antibody (Abcam) diluted to 1:50 in Bond Primary Diluent in a humid chamber at 40°C overnight. Following washes with PBS, all of the TMAs were incubated for 30 min with secondary antibody (GTVision™III Detection System/Mo&Rb) at RT. The sections were then counterstained with Gill hematoxylin and mounted after clearing with xylene. The negative controls were a group of the same TMAs subjected to the same procedures, excluding the primary antibodies.

### Staining evaluation

Immunostaining was independently reviewed by two pathologists (TC and SJ) who were blinded to patient characteristics and outcome. A staining index (SI) was calculated according to the intensity and percentage of positive cells. A semiquantitative grading system incorporating staining intensity (0, no staining; 1, weak; 2, moderate; 3, strong) and the percentage of cells stained (0, no staining; 1, < 10%; 2, 10–50%; and 3, > 50% of tumor cells) was applied. The SI was calculated by multiplying the results of these two variables and ranged from 0 to 9 [32, 33]. If heterogeneity was observed for the 2 cylinders of each case, the SI was defined as positive or negative so that one overall mean score was used. A cutoff representing the upper quartile (SI > 4) was used to define high levels of staining, whereas others were defined as low levels of staining. The SI for each antibody was evaluated using the same criteria.

### Statistical analysis

DFS duration was defined as the interval from initial surgery to a clinically defined metastasis. Pearson's χ^2^ or Fisher's exact tests were used to evaluate the significance of differences between the covariates. Postoperative DFS probability was determined using the Kaplan-Meier method, and differences in survival between markers were estimated using 2-sided log-rank (Mantel-Cox) tests. Data were analyzed using SPSS (version 20.0; SPSS Inc.). All *P* values are two-sided, and a *P* value of less than 0.05 was considered statistically significant.

For the predictive model, variables with *P* values less than 0.2 with a univariate analysis of the training set were used to construct the multivariate Cox proportional hazards models. To estimate the utility of the prognostic model, the area under the receiver operating curve (ROC) for patient DFS was calculated. The time-dependent ROC curve was used to illustrate the relationship between the sensitivity and false-positive rate (1-specificity) We calculated a risk score of each patient by the formula and used time-dependent ROC analysis by R software version 3.1.1 and the “survival ROC” package to assess the prognostic accuracy of the score. Then we used Youden index to choose the best cutoff score based on the ROC curve.

## SUPPLEMENTARY FIGURE AND TABLES




