# Supplementary material for: Stathmin and phospho-stathmin protein signature is associated with survival outcomes of breast cancer patients
Source: Oncotarget. 2015 Jun 13;6(26):22227–38. doi: 10.18632/oncotarget.4276 (PMC4673159; doi:10.18632/oncotarget.4276)
Supplement: Supplementary file 1 [file oncotarget-06-22227-s001.pdf]

SUPPLEMENTARY FIGURE AND TABLES

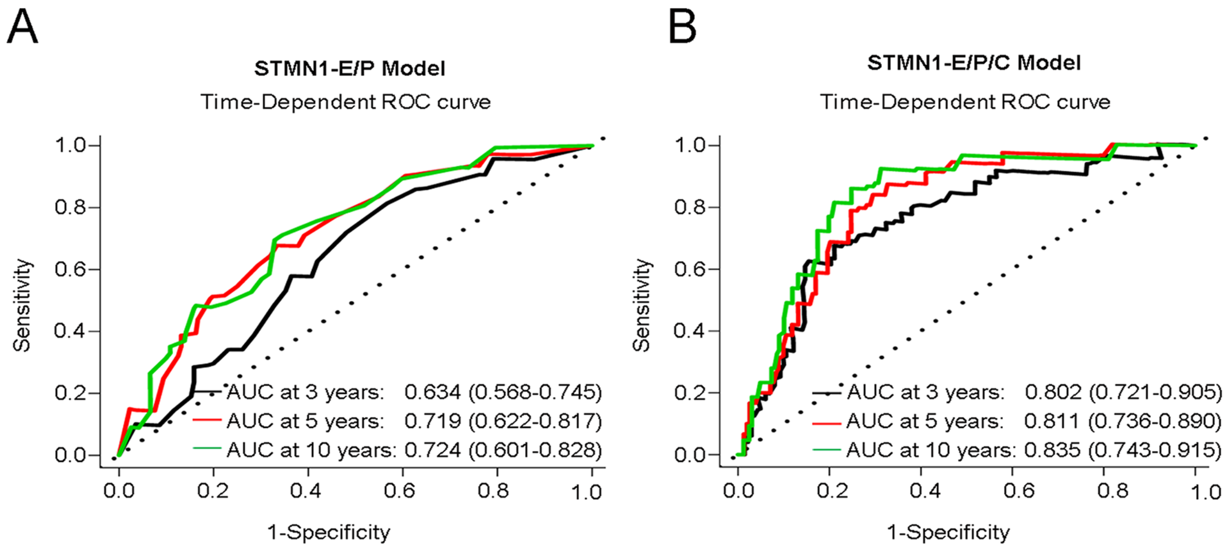

Supplementary Figure S1: Time-dependent ROC curves for patients in the training set. We used AUCs at 3, 5 and 10 years according to the STMN1-E/P and STMN1-E/P/C models, respectively, to assess prognostic accuracy and calculated *P* values using the log-rank test.

Supplementary Table S1: Clinicopathological characteristics and the expression of STMN1, Ser16, Ser25, Ser38 and Ser63 in both training cohort and validation cohort

Supplementary Table S2: Clinicopathological characteristics and the risk score derived by the STMN1-E/P model in both training set and validation set

Characteristics	Training set (*n* = 204)			Validation set (*n* = 106)		
	Low risk	High risk	*P*[a]	Low risk	High risk	*P*[a]
Age			0.888			0.050
<50 y	42 (20.6%)	49 (24.0%)		18 (17.1%)	34 (10.0%)	
≥50 y	54 (26.5%)	59 (28.9%)		29 (27.6%)	24 (22.9%)	
NA	0 (0.0%)			1 (0.9%)		
Menopausal status			1.000			0.694
Premenopausal	40 (19.6%)	44 (21.6%)		19 (17.9%)	27 (25.5%)	
Postmenopausal	56 (27.5%)	64 (31.4%)		28 (26.3%)	32 (30.2%)	
Tumor stage			0.631			0.181
I	26 (12.8%)	33 (16.3%)		14 (13.3%)	18 (17.1%)	
II	54 (26.6%)	53 (26.1%)		25 (23.8%)	22 (21.0%)	
III	16 (7.9%)	21 (10.3%)		8 (7.6%)	18 (17.1%)	
NA	1 (0.5%)			1 (0.9%)		
Histological grade			**0.006**			**0.046**
I & II	81 (39.7%)	73 (35.8%)		43 (40.6%)	37 (34.9%)	
III	15 (7.4%)	35 (17.2%)		8 (7.5%)	18 (17.0%)	
Tumor size			**0.000**			**0.000**
<2 cm	63 (30.9%)	30 (14.7%)		42 (39.6%)	19 (17.9%)	
≥2 cm	39 (19.1%)	72 (35.3%)		9 (8.5%)	36 (34.0%)	
Node status			**0.033**			**0.000**
Negative	48 (23.8%)	69 (34.2%)		48 (45.3%)	33 (31.1%)	
Positive	48 (23.8%)	37 (18.3%)		3 (2.8%)	22 (20.8%)	
NA	1 (0.5%)			0 (0.0%)		
ER status			0.777			**0.000**
Negative	43 (21.4%)	51 (25.4%)		16 (15.2%)	43 (41.0%)	
Positive	52 (25.9%)	55 (27.4%)		30 (28.6%)	16 (15.2%)	
NA	3 (1.5%)			1 (0.9%)		
PR status			0.550			**0.000**
Negative	59 (29.5%)	73 (36.5%)		20 (19.0%)	46 (43.8%)	
Positive	34 (17.0%)	34 (17.0%)		26 (24.8%)	13 (12.4%)	
NA	4 (2.0%)			1 (0.9%)		
HER2 status			0.262			0.630
Negative	53 (26.2%)	51 (25.2%)		35 (33.3%)	48 (45.7%)	
Positive	42 (20.8%)	56 (27.7%)		11 (10.5%)	11 (10.5%)	
NA	2 (1.0%)			1 (0.9%)		

Abbreviations: *ER*, estrogen receptor; *PR*, progesterone receptor; *HER2*, human epidermal growth factor receptor 2; *NA*, not available

Bold values are significant ($P < 0.05$).

[a]Based on Pearson's χ^2 test

Supplementary Table S3: Clinicopathological characteristics and the risk score of STMN1-E/P/C model in both training set and validation set

Characteristics	Training set (*n* = 204)			Validation set (*n* = 106)		
	Low risk	High risk	*P*[a]	Low risk	High risk	*P*[a]
Age			0.573			0.243
<50 y	43 (21.1%)	48 (23.5%)		22 (21.0%)	30 (28.6%)	
≥50 y	59 (28.9%)	54 (26.5%)		29 (27.6%)	24 (22.9%)	
NA	0 (0.0%)			1 (0.9%)		
Menopausal status			0.670			1.000
Premenopausal	40 (19.6%)	44 (21.6%)		22 (20.8%)	24 (22.6%)	
Postmenopausal	62 (30.4%)	58 (28.4%)		29 (27.4%)	31 (29.2%)	
Tumor Stage			**0.000**			**0.000**
I	48 (23.6%)	11 (5.4%)		23 (21.9%)	9 (8.6%)	
II	46 (22.7%)	61 (30.0%)		25 (23.8%)	22 (21.0%)	
III	7 (3.4%)	30 (14.8%)		3 (2.9%)	23 (21.9%)	
NA	1 (0.5%)			1 (0.9%)		
Histological grade			**0.000**			**0.046**
I & II	92 (45.1%)	62 (30.4%)		43 (40.6%)	37 (34.9%)	
III	104.9%)	40 (19.6%)		8 (7.5%)	18 (17.0%)	
Tumor size			**0.000**			**0.022**
<2 cm	63 (30.9%)	30 (14.7%)		31 (28.6%)	19 (18.1%)	
≥2 cm	39 (19.1%)	72 (35.3%)		21 (20.0%)	35 (33.4%)	
Node status			**0.000**			**0.000**
Negative	78 (38.4%)	39 (19.2%)		42 (39.6%)	19 (17.9%)	
Positive	23 (11.3%)	63 (31.0%)		9 (8.5%)	36 (34.0%)	
NA	1 (0.5%)			0 (0.0%)		
ER status			0.323			**0.019**
Negative	43 (21.4%)	51 (25.4%)		22 (21.0%)	37 (35.2%)	
Positive	57 (28.4%)	50 (24.9%)		28 (26.7%)	18 (17.1%)	
NA	3 (1.5%)			1 (0.9%)		
PR status			0.551			0.225
Negative	63 (31.5%)	69 (34.5%)		28 (26.7%)	38 (36.2%)	
Positive	36 (18.0%)	32 (16.0%)		22 (21.0%)	17 (16.2%)	
NA	4 (2.0%)			1 (0.9%)		
HER2 status			0.324			0.483
Negative	48 (23.8%)	56 (27.7%)		38 (36.2%)	45 (42.9%)	
Positive	53 (26.2%)	45 (22.3%)		12 (11.4%)	10 (9.5%)	
NA	2 (1.0%)			1 (0.9%)		

Abbreviations: *ER*, estrogen receptor; *PR*, progesterone receptor; *HER2*, human epidermal growth factor receptor 2; *NA*, not available

Bold values are significant ($P < 0.05$).

[a]Based on Pearson's χ^2 test
